# Criterion validity and test-retest reliability of SED-GIH, a single item question for assessment of daily sitting time

**DOI:** 10.1186/s12889-018-6329-1

**Published:** 2019-01-05

**Authors:** Kristina Larsson, Lena V. Kallings, Örjan Ekblom, Victoria Blom, Eva Andersson, Maria M. Ekblom

**Affiliations:** The Swedish School of Sport and Health Sciences (GIH), Box 5626, 11486 Stockholm, Sweden

**Keywords:** activPAL, Adults, Office-based work, Older adults, Reliability, Sedentary behaviour, Sitting, Validity

## Abstract

**Background:**

Sedentary behaviour has been closely linked to metabolic and cardiovascular health and is therefore of importance in disease prevention. A user-friendly tool for assessment of sitting time is thus needed. Previous studies concluded that the present tools used to assess a number of sedentary behaviours are more likely to overestimate sitting than single-item questions which often underestimate sitting time, and that categorical answering options are recommended. In line with this, the single-item question with categorical answering options, SED-GIH, was developed.

The aim of this study was to investigate the criterion validity of the SED-GIH question using activPAL3 micro as the criterion measure. The second aim was to evaluate the test-retest reliability of the SED-GIH questionnaire.

**Method:**

In the validity section of this study, 284 middle-aged adults answered a web questionnaire, which included SED-GIH, wore activPAL and filled in a diary log for one week. Spearman’s rho assessed the relationship between the SED-GIH answers and the daily average sitting time as monitored by the activPAL (activPAL-SIT), a Weighted Kappa assessed the agreement, ANOVA assessed differences in activPAL-SIT between the SED-GIH answer categories, and a Chi^2^ compared the proportions of hazardous sitters between the different SED-GIH answer categories. In the reliability section, 95 elderly participants answered the SED-GIH question twice, with a mean interval of 5.2 days. The reliability was assessed with ICC and a weighted Kappa.

**Results:**

The SED-GIH question correlated moderately with activPAL-SIT (rho = 0.31), with a poor agreement (weighted Kappa 0.12). In total, 40.8% underestimated and 22.2% overestimated their sitting time. The ANOVA showed significant differences in activPAL-SIT between the different SED-GIH answer categories (*p* < 0.001). The Chi^2^ showed a significant difference in proportion of individuals sitting more than 10 h per day within each SED-GIH answer category. ICC for the test-retest reliability of SED-GIH was excellent with ICC = 0.86, and the weighted Kappa showed an agreement of 0.77.

**Conclusions:**

The unanchored single item SED-GIH question showed excellent reliability but poor validity in the investigated populations. Validity and reliability of SED-GIH is in line with other questionnaires that are commonly used when assessing sitting time.

## Background

Sedentary behaviour is being increasingly recognized as a health risk for all-cause mortality, cancer, cardiovascular diseases, type 2 diabetes and metabolic risks, even if the recommendations for physical activity are fulfilled [1, 2]. Individuals sitting ≥10 h per day have an increased risk of all-cause mortality and cardiovascular disease, based on subjective measures [3, 4]. With objective measures, risk of mortality was observed at a sitting level of ≥8.2 h per day [5]. Sedentary behaviour is defined as waking behaviour while in a sitting, reclining or lying posture, with an energy expenditure ≤1.5 metabolic equivalents (METs) [6].

At present, both objective and subjective measurement methods are commonly used to assess sitting time. The most accurate method and gold standard is direct observation, which is a valid criterion measure [7]. Since direct observation can be difficult to use when observing large groups of people, other methodological devices have been developed. One is activPAL3, a triaxial accelerometer assessing sitting by monitoring the positioning of a limb in relation to the horizontal plane. The activPAL3 is usually worn on the midline of the anterior aspect of the thigh and identifies episodes of walking, sitting and standing by measuring accelerations in three planes [8]. The activPAL3 has been extensively validated in several populations [9]. In adults, a validation study of activPAL3 data, which was compared to video observation showed an agreement of 97% in activities of daily living [10]. The activPAL3 is often used as a criterion measurement when validating other objective and subjective measurement methods for sedentary behaviour [11]. The most common subjective measurement methods for assessing sedentary behaviour are self and proxy-report questionnaires, behaviour logs, short-term recall and diaries. General limitations with subjective methods include recall and reporting bias, and random and systematic reporting errors yielding low validity [12, 13]. The newly developed TAxonomy of Self-reported Sedentary behaviour Tools (TASST) framework investigated the validity of 32 different self-reporting tools against objective measurement methods. These tools are categorized into four domains: type of assessment, recall period, temporal unit and assessment period. According to TASST, all tools reported poor accuracy with underestimations ranging from 1.1 to 6 h, and overestimations of up to 2.2 h of sedentary behaviour. Tools assessing a sum of sedentary behaviours using a composite of several items were more likely to overestimate sitting than single-item questionnaires [14]. Regarding physical activity, categorical answering options have been shown to have some advantages over open answer alternatives [15]. To the author’s knowledge, a categorical single item question assessing sedentary behaviour has not been validated. Therefore, a new single item question with categorical answering options, SED-GIH, was developed (taxon 1.1.1/2.4/3.1/4.5).

The aim of the current study was to investigate the validity of the SED-GIH question using activPAL3 micro as the criterion measure. The second aim was to evaluate the test-retest reliability of the SED-GIH questionnaire.

## Method

### Participants and sampling

The data in this study were retrieved from two larger projects with cross-sectional and cohort designs, respectively. The Stockholm regional ethical review board approved both projects, Dnr 2016/796–31 and Dnr 2014/1526–32, respectively. All participants signed a written informed consent form prior to participating.

## Criterion validity

The validity section of this study was a part of the research project “Physical Activity and Healthy Brain Functions” performed at The Swedish School of Sport and Health Sciences (GIH). The project was carried out during 2016–2017. Participants were recruited through convenience sampling at two office work sites in Stockholm and Gothenburg. The inclusion criteria were employees with an office-based job at these companies. A total of 1971 employees were invited to participate via mail, of which 284 provided complete data and were included in the analysis. Initially, participants responded to a self-reported web questionnaire, which included the SED-GIH question and demographic information about age, gender and education. Approximately two weeks later (mean 16 days ±14 days), the participants attended a test session where they were equipped with the activity monitor activPAL3 micro (from now on referred to as activPAL) by a test leader and received a diary log. The participants wore the activPAL for seven consecutive days, 24 h a day. During the same period, they noted the time points when they went to bed and woke up in the diary log. After the measuring period, participants returned the activPAL and the diary log to a mailbox, which was emptied by the test leader.

## Test-retest reliability

The reliability section of this study was provided with data from the “Health Project” at GIH. The project was carried out during 2016. The project is a collaboration between GIH and the municipalities of Solna and Lidingö in Stockholm. The municipalities informed potential participants about the project, which they then voluntarily signed up for. The inclusion criterion for this sample was elderly individuals ≥65 years old. The participants attended two test sessions with a mean of 5.2 days (min = 1 day, max = 16 days) in between. In each test session they responded to a self-reported paper questionnaire, which included the SED-GIH question and demographic information about age and gender. The questionnaire was handed out by a test leader, filled in by the respondent and then directly returned to the test leader on each test occasion.

### Outcome measures

## SED-GIH

The single item question SED-GIH reads “How much time do you sit during a normal day, excluding sleep?” There are seven categorical answer options: “Virtually all day”, “13–15 h”, “10–12 h”, “7–9 h”, “4–6 h”, “1–3 h” and “Never”. According to TASST, SED-GIH is defined as a single item direct measure of sitting, for an unanchored recall period with a temporal unit of a day, and a non-defined assessment period (taxon 1.1.1/2.4/3.1/4.5) [14]. The question was used in two formats, a web questionnaire (validity data) and a paper questionnaire (reliability data). The categorical answer options of the SED-GIH question was recoded from 1 to 7, with 1 corresponding to the answer “Never” and 7 to the answer “Virtually all day”.

## activPAL

The criterion measure for this study was sitting time as measured by the triaxial activPAL3 micro (PAL Technologies Ltd., Glasgow, Scotland) activity monitor. To waterproof the activPAL, it was placed in a small condom with transparent film around (Tegaderm Roll, 3 M), which also was used by the test leader to attach the activPAL onto the frontal aspect of the midline of the participant’s right thigh. The activPAL continuously recorded orientation of the thigh at a sampling rate of 20 Hz. After analysis of raw data (activPAL software version 7.2.32), periods of sitting/lying, standing and walking were identified. The original data from activPAL consisted of one file for each participant containing data from all seven consecutive days. Excel HSC PAL analysis software V2 19 s, developed by Dr. Philippa Dall and Professor Malcolm Granat, School of Health and Life Sciences, Glasgow Caledonian University, was used to merge the file with the time parameters from the diary log. For missing values in the diary log, standardised times were used (wakening time 6 am, bedtime 11 pm). If bedtime was later than midnight, a separate analysis was conducted on the following date for the time spent awake past midnight. These hours were added to the correct date afterwards. The data were categorised as: total wear time, sitting time, standing time and walking time.

Inclusion criterion was at least four total consecutive days. The day the activPAL was attached on the participants was always excluded, since it was not a presentable 24-h day. The daily average activPAL sitting time was calculated as a continuous variable and is from now on referred to as activPAL-SIT.

### Statistical analysis

Analysis was conducted using IBM SPSS Statistics version 24 and Microsoft Excel 2013 using Real Statistics Resource Pack. The level of statistical significance was set at *p* < 0.05. Since very few participants chose the answer options “never” and “virtually all the time” in the SED-GIH, the data were recategorised from seven into five categories (merging “Never” and “1–3 h” to “≤ 3 hours”, and “13–15 h” and “Virtually all day” to “≥ 13 hours”). The continuous activPAL-SIT data were categorized into the same five categories as the SED-GIH categorical answer options. The activPAL-SIT data categories were coded as 1 = ≤ 3 h, 2 = 4–6 h, 3 = 7–9 h, 4 = 10–12 h and 5 = ≥ 13 h.

## Criterion validity

Correlation between SED-GIH and activPAL-SIT were assessed using Spearman’s rho with 95% confidence interval (CI). The associations were interpreted as weak (Spearman’s rho < 0.10), modest (Spearman’s rho 0.1–0.3), moderate (Spearman’s rho 0.3–0.5), strong (Spearman’s rho 0.5–0.8) or very strong (Spearman’s rho 0.8–1.0) [16]. To assess the agreement between SED-GIH and the categorized activPAL-SIT, a weighted Kappa was conducted. The agreement was interpreted as poor (Kappa < 0.20), fair (Kappa 0.21–0.40), moderate (Kappa 0.41–0.60), substantial (Kappa 0.61–0.80) or almost perfect (Kappa 0.81–1.00) [17]. The categorized activPAL-SIT was used in the Spearman’s rho and the weighted Kappa analysis. To assess the distribution of over and underestimations of sitting time, calculations in Excel were conducted. A one-way independent ANOVA with subsequent post-hoc Tukey test was used to assess whether any differences existed in the continuous activPAL-SIT data between the different SED-GIH answer categories. Further, we dichotomized the continuous activPAL-SIT data at 10 h per day, and participants were divided into high (≥ 10 h) or low (< 10 h) sitting, based on this. Those in the ≥10 h group were defined as hazardous sitters. A Chi^2^ analysis was then performed to compare proportions of hazardous sitters between the different SED-GIH answer categories. Sensitivity and specificity analysis was used to identify the proportion of true positive and true negative answers of SED-GIH, based on the dichotomized activPAL-SIT data.

## Test-retest reliability

Intra-class correlation (ICC) was calculated to assess the test-retest reliability of the SED-GIH. The intra-class correlation coefficients were interpreted as poor (ICC < 0.40), fair (ICC 0.40–0.59), good (ICC 0.60–0.74) or excellent (ICC 0.75–1.00) [18]. A Weighted Kappa was conducted to assess the agreement between the test and the retest. The agreement was interpreted as described above [17].

## Results

### Characteristics validity sample

**Fig. 1:**
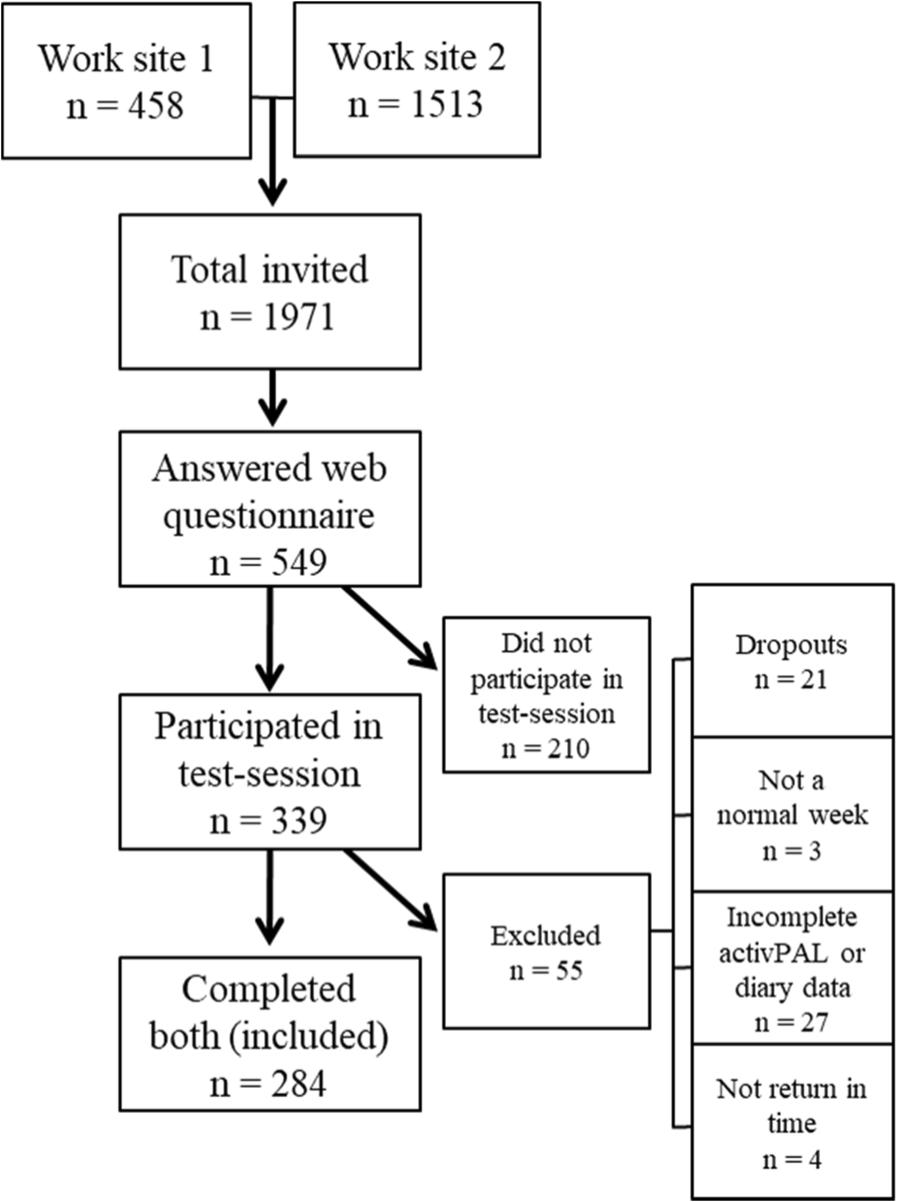
Flow-chart of the number of participants in “Physical Activity and Healthy Brain Functions” project

A total number of 284 participants (95 males, 188 females, 1 missing gender) with a mean age (SD) of 42.9 (8.9) years were included in the validity part of this study. In the study population, 2.1% had secondary school as their highest education level, 38.7% upper secondary school education, 54.2% higher education and 4.2% had a postgraduate education. The number of participants from each company and number of participants that fulfilled both the test session and the web questionnaire are presented in Fig. 1.

The activPAL was worn for a mean (SD) of 6.5 (0.5) days and a mean awake wear time (SD) of 16.3 (0.7) hours per day. The disposition of the number of participants in each categorical answer option of the SED-GIH question is presented in Table 1. The mean hours of sitting per day (SD), measured by activPAL, were 9.7 (1.4). Table 2 presents the number of participants in each categorized activPAL-sit group.

**Table 1:** The disposition of participants’ answers of SED-GIH question and their mean hours of sitting per day as measured with activPAL

SED-GIH answer categories	n	% of total n	activPAL, mean hours of sitting time per day	95% Confidence Interval	
				Lower	Upper
≥ 13 h ^a, c^	35	12.3	10.3	9.7	10.8
10–12 h ^b, d^	84	29.6	10.1	9.8	10.5
7–9 h	102	35.9	9.6	9.3	9.9
4–6 h ^c, d^	45	15.8	9.2	8.8	9.6
≤ 3 h ^a, b^	18	6.3	8.7	8.1	9.3
Total	284	100	9.7	9.6	9.9

Note. ^a^indicates significant difference (*p* < 0.001) between group **≥**13 h and ≤ 3 h

^b^indicates significant difference (*p* < 0.001) between group 10–12 h and ≤ 3 h

^c^indicates significant difference (*p* < 0.05) between group **≥**13 h and 4–6 h

^d^indicates significant difference (*p* < 0.05) between group 10–12 h and 4–6 h

**Table 2:** The disposition of the number of participants in each categorized activPAL-SIT group

activPAL categories	n	% of total n
≥ 13 h	6	2.1
10–12 h	153	53.9
7–9 h	121	42.6
4–6 h	4	1.4
≤ 3 h	0	0
Total	284	100

### Characteristics reliability sample

A total number of 221 elderly persons participated in the “Health project”. In the first test session, 172 answered the questionnaire with SED-GIH included. A total of 95 (29 males, 66 females) answered the questionnaire at both the first and second test session and were therefore included in the reliability part of this study. The participants’ mean age (SD) was 70.3 (5.0) years.

### Criterion validity

The correlation between the SED-GIH answer categories and continuous activPAL-SIT for all days was significant (*p* < 0.001) and moderate with Spearman’s rho = 0.31 (CI = 0.20–0.41). The weighted Kappa showed a poor agreement of 0.12 (CI = 0.05–0.18) between the SED-GIH answer categories and categorized activPAL-SIT. Table 3 presents the distribution of the number of participants in each activPAL-SIT data category and each SED-GIH answer category. The proportion of participants that underestimated their sitting time was almost twice that of the proportion that overestimated their sitting time. In total, 105 participants (corresponding to 37.0% of the total) estimated their sitting correctly, 116 (40.8%) underestimated their sitting and 63 (22.2%) overestimated their sitting. The 7–9 and 10–12 h spent sitting groups presented the highest numbers of correct estimations with 47.1 and 64.3%, respectively.

**Table 3:** The relationship between the number of participants in each activPAL-SIT data category and in each SED-GIH answer category

activPAL-SIT category, n (% of correct estimations)						
SED-GIH answer categories, n	≤ 3 hours	4-6 hours	7-9 hours	10-12 hours	≥ 13 hours	Total
≤ 3 hours	0 (0)	0	13	5	0	18
4-6 hours	0	1 (2.2)	23	20	1	45
7-9 hours	0	2	48 (47.1)	51	1	102
10-12 hours	0	1	27	54 (64.3)	2	84
≥ 13 hours	0	0	10	23	2 (5.7)	35
Total	0	4	121	153	6	284

Table 3 shows that participants who chose the answer ≤3 h in the SED-GIH question all underestimated their sitting time as compared to the categorical values of activPAL-SIT. Furthermore, nearly all participants who chose ≥13 h overestimated their sitting time compared to activPAL-SIT.

A one-way independent ANOVA comparing activPAL-SIT between individuals in the five SED-GIH categories showed that significant differences in mean activPAL-SIT existed between the categories (*p* < 0.001). Significant differences between the SED-GIH categories are presented in Table 1.

Cross-tabulating the SED-GIH answer categories with dichotomized continuous activPAL-SIT data at 10 h per day identified the proportion of high and low sitting in the SED-GIH strata. The Chi^2^ test showed that there was a significant difference (p < 0.001) between the proportion of individuals sitting more or less than 10 h, when comparing SED-GIH and activPAL-SIT. Results are displayed in Table 4 and show that among individuals rating themselves as sitting ≥10 h using the SED-GIH questionnaire, the majority (56.3%) were sitting more than 10 h per day according to the activPAL-SIT. However substantially lower proportions of hazardous sitting (29.7%) was seen among individuals who rated themselves as sitting < 10 h. Still, the ability of SED-GIH to classify individuals sitting ≥10 h was rated as poor, with a sensitivity of 58% and a specificity of 69%.

**Table 4:** The proportion of individuals sitting for more, or less, than 10 h per day based on activPAL-SIT

Actual sitting time as measured by activPAL-SIT	SED-GIH answer categories		
	< 10 h	≥ 10 h	Total
*n* < 10 h (% of total n)	116 (70.3)	52 (43.7)	168 (59.2)
n ≥ 10 h (% of total n)	49 (29.7)	67 (56.3)	116 (40.8)
Total n	165	119	284

### Test-retest reliability

The number of participants in each SED-GIH answer category at test sessions one (T1) and two (T2) is presented in Table 5. Intra-class correlation coefficient for the test-retest reliability of the SED-GIH question answers was excellent with ICC = 0.86 (CI = 0.79–0.90). The weighted Kappa showed substantial agreement of 0.77 (CI = 0.68–0.86) between the two SED-GIH question answering occasions.

**Table 5:** The number of the participant answers at SED-GIH test sessions one (T1) and two (T2)

T2 answer categories, n						
T1 answer categories, n	≤ 3 hours	4-6 hours	7-9 hours	10-12 hours	≥ 13 hours	Total
≤ 3 hours	9	6	0	0	0	15
4-6 hours	1	38	5	1	0	45
7-9 hours	0	2	21	1	0	24
10-12 hours	0	0	1	6	1	8
≥ 13 hours	0	0	0	2	1	3
Total	10	46	27	10	2	95

## Discussion

The aim of the current study was to investigate the criterion validity and test-retest reliability of the SED-GIH question using activPAL3 micro as the criterion measure. The main findings were a moderate correlation (*r* = 0.31, CI = 0.20–0.41) and a poor agreement (weighted Kappa 0.12, CI = 0.05–0.18) between SED-GIH and activPAL derived sitting time (activPAL-SIT). Significant differences in activPAL-SIT existed between individuals in the different categorical answer options of SED-GIH. The reliability of SED-GIH was excellent (ICC = 0.86, CI = 0.79–0.90) with a substantial agreement (weighted Kappa 0.77, CI = 0.68–0.86).

The TASST framework was developed to gain an overview of tools used for assessing sedentary behaviour, and categorized them into four domains: type of assessment, recall period, temporal unit and assessment period. According to TASST, SED-GIH is defined as a single item direct measure of sitting, for an unanchored recall period with a temporal unit of a day, and an non-defined assessment period (taxon 1.1.1/2.4/3.1/4.5) [14]. The moderate correlation between sitting time measured objectively with activPAL and sitting time measured subjectively using the SED-GIH question is in line with other questionnaires. IPAQ (International Physical Activity Questionnaire, (TASST taxon 1.1.1/2.2/3.1/4.3) contains three specific sitting items, which have been validated using activPAL. For sitting time during weekdays, including transportation, correlation was low (*r* = 0.16, ICC = 0.15) and non-significant (*p* = 0.2) between the two methods. Here, IPAQ underestimated sitting time by 2.2 h per day [19]. PAST (Past-day Adults Sedentary Time, TASST taxon 1.2.2.1/2.1/3.1/4.5) and PAST-U (modified version of PAST, TASST taxon 1.2.2.1/2.1/3.1/4.5) asks participants to report their time spent sitting or lying during the previous day. When using activPAL (version 3) as criterion measure, the validity for PAST was assessed to be *r* = 0.57 [20], and PAST-U ICC = 0.64 [21]. When Busschaert and co-workers tested the validity of three different questionnaires measuring context-specific sedentary behaviour (TASST taxon 1.2.2.1/2.2/3.1/4.3, 1.2.2.1/NA/NA/NA, 1.2.2.1/2.4/3.1/4.3) they found weak to acceptable validity for adults (*r* = 0.06–0.52) and older adults (*r* = 0.38–0.50) [22]. This implies that the SED-GIH has stronger associations with objective sitting than other single item questionnaires, such as IPAQ, when compared to direct measurement. However, these associations are not as strong as the time-specified PAST and PAST-U, which collect information on sitting during the previous day only.

Participants who estimated their sitting as ≤3 h using SED-GIH, all underestimated their sitting time as compared to activPAL-SIT (see Table 3). Furthermore, participants who estimated their sitting as ≥13 h almost all overestimated their sitting time. These results are in line with comparisons between PAST and activPAL (version 3) derived sitting times. PAST underestimated sitting times at low levels of sitting, and overestimated sitting time at high levels of sitting [20]. However, a Bland Altman between IPAQ and activPAL indicated that IPAQ underestimated sitting time by up to 2.2 h per day (during a total week including transportation) [19], and both PAST-U and the three different questionnaires measuring context-specific sedentary behaviour overestimated sedentary time, with activPAL as the criterion measure [21, 22]. Dall and colleagues concluded that most sitting questionnaires underestimate sitting time by 2–4 h per day. Single item questionnaires are more likely to underestimate sitting time, while questionnaires assessing sitting during a sum of sedentary behaviours using a composite of several items tend to overestimate sitting time. Questionnaires assessing sitting during a sum of sedentary behaviours over an unanchored or longer period of time tend to report larger underestimations [14]. According to this study, the reasons for sitting time underestimations by the SED-GIH question can be explained by it being based on a single-item question during an unanchored period of time.

The original seven SED-GIH answer categories were collapsed into five, since there were very few participants choosing “Virtually all day” or “Never”. The intention of including all seven answering options was that “Virtually all day” and “Never” might be easier to relate to instead of < 1 h and > 15 h. They also provide the answer options with some anchorage. When the five categories were analysed, the mean values (displayed in Table 1) of sitting time measured with activPAL did not differ much between the categorical answer options of SED-GIH (varying from 8.7 to 10.3 h per day, mean 9.7 h per day). Thus, the objectively measured average sitting time per day had a narrow distribution, even though the participants subjectively estimated their sitting time with SED-GIH in a wider range. However, the accuracy of SED-GIH changed when only two categories were used (more or less than 10 h of sitting per day). The majority of the participants who rated themselves as sitting for 10 h or more, actually sat for more than 10 h (56.3%). The low sensitivity and specificity of SED-GIH indicates that it would not be useful for identifying hazardous sitters (≥ 10 h per day). Objective measurements may be more useful in detecting sedentary behaviour, possibly in combination with PAST or similar questionnaires. More research is thus needed to develop questionnaires assessing sedentary behaviour and provide better outcomes together with objective methods.

Test-retest reliability of SED-GIH was excellent (ICC = 0.86, CI = 0.79–0.90), which is better than other reliability tested questionnaires. PAST had fair to good reliability (ICC = 0.50), and three different questionnaires measuring context-specific sedentary behaviour had good reliability for adults (ICC = 0.73–0.77) and older adults (ICC = 0.68–0.80) [20, 22]. However, SED-GIH is a single item questionnaire, whereas PAST and the three different questionnaires measuring context-specific sedentary consist of several questions, which can affect test-retest reliability. With a tool consisting of a single item question, it might be easier to answer the same question twice compared to tools consisting of several questions. Thus, SED-GIH has good repeatability and generates reliable answers among older adults. However, it is not known whether SED-GIH can detect changes of sedentary behaviour over time, such as before and after a behavioural change intervention period. This field needs further research.

Limitations to the current study have been observed in the methods and the processing of the data. Participants may have become more conscious about their habits regarding sitting time when they answered the web questionnaire prior to the objective measures, which may have affected their sitting habits during the week of measurement with activPAL. Additionally, the measurement period between answering SED-GIH and wearing the activPAL varied (mean 16 days ±14 days), which may have affected the agreement. One impact on internal validity is the accuracy of the participants’ dedication to fill in the diary log correctly, which can affect the whole dataset. In the validity study, participants were employees with an office-based job, which is not representative of a general population. SED-GIH should be validated in other contexts and with different populations. In the reliability part of the current study, all participants were elderly. This may have an effect on the results since some elderly persons can have reduced memory function compared to younger adults.

### Implications

SED-GIH may be useful as a tool when identifying sitting time as a determinant for health risks on a population level, but would not in itself be sufficiently informative for screening for unhealthy sitting habits in primary care. More studies performed on a broader population are needed.

## Conclusion

The unanchored single item SED-GIH question showed excellent reliability but poor validity in the investigated populations.

## Abbreviations

activPAL-SIT: activPAL defined daily sitting time
ANOVA: Analysis of variance
CI: Confidence interval
GIH: The Swedish School of Sport and Health Sciences
Hz: Hertz
ICC: Intra-class correlation coefficient
IPAQ: International Physical Activity Questionnaire
MET: Metabolic equivalent
p: probability
PAST: Past-day Adults Sedentary Time
PAST-U: Modified version of PAST that asks participants to report their time spent sitting or lying during the previous day
SD: Standard deviation
SED-GIH question: The newly developed single item question on habitual sedentary behaviour
TASST: TAxonomy of Self-reported Sedentary behaviour Tools
